# Supplementary material for: Three-dimensional open architecture enabling salt-rejection solar evaporators with boosted water production efficiency
Source: Nat Commun. 2022 Nov 4;13:6653. doi: 10.1038/s41467-022-34528-7 (PMC9636182; doi:10.1038/s41467-022-34528-7)
Supplement: Supplementary file 1 — Supplementary Information [file 41467_2022_34528_MOESM1_ESM.pdf]

Supplementary Information for

Three-dimensional open architecture enabling salt-rejection solar evaporators with boosted water production efficiency

Kaijie Yang,[1,†] Tingting Pan,[1,†] Saichao Dang,[2] Qiaoqiang Gan [2,*] and Yu Han [1,*]

[1]Advanced Membranes and Porous Materials (AMPM) Center, Physical Sciences and Engineering Division, King Abdullah University of Science and Technology (KAUST), Thuwal, Saudi Arabia

[2]Materials Science Engineering Program, Physical Science and Engineering Division, King Abdullah University of Science and Technology (KAUST), Thuwal, Saudi Arabia

[†]Authors contributed equally.

*Corresponding author.

E-mail address: qiaoqiang.gan@kaust.edu.sa (Q. Gan)

yu.han@kaust.edu.sa (Y. Han).

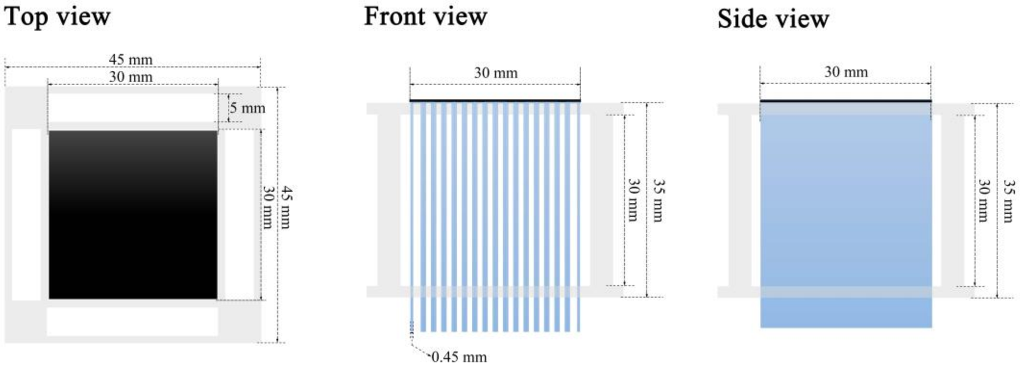

Figure S1. Size information of the prototype. The PMMA frame is used for support.

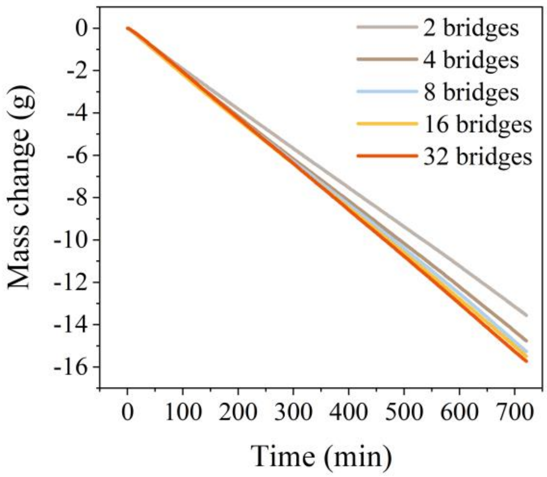

Figure S2. Mass change curves of evaporators with different bridge numbers when processing 10 wt.% NaCl solution under 1 sun illumination.

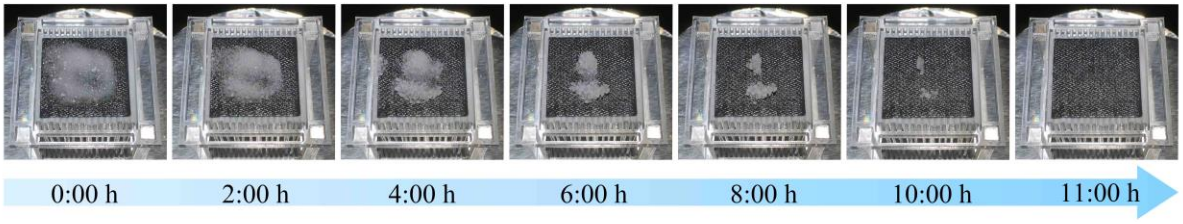

Figure S3. Progression of salt redissolving from the 32-bridge evaporator surface. The solar intensity is 1 sun. The saline water concentration is 10 wt.% NaCl.

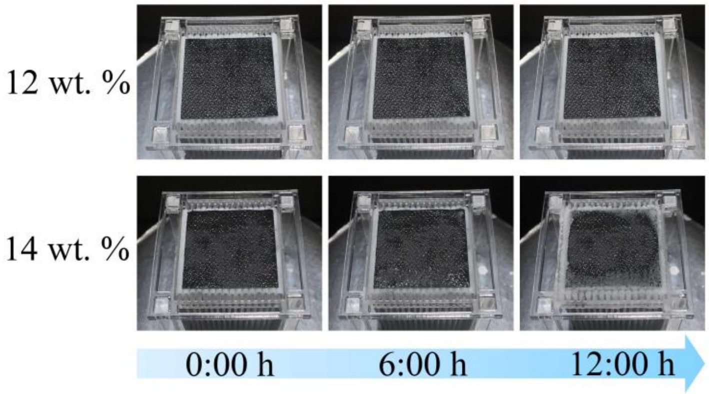

Figure S4. Photographic recording of the salt accumulation on the evaporator surface when processing 12 and 14 wt.% NaCl solutions.

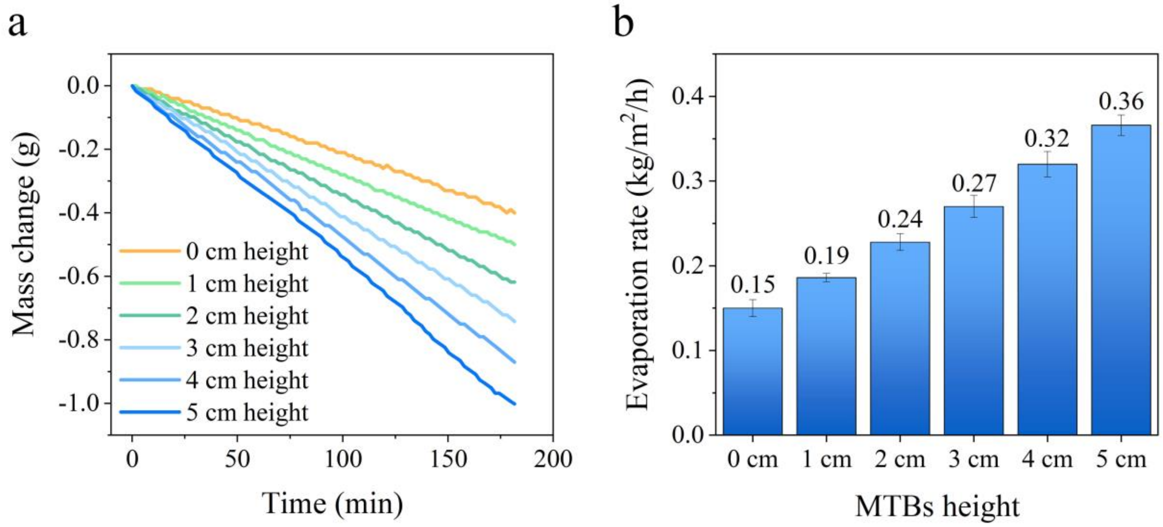

Figure S5. Evaporation performance of evaporators with different MTB heights under a dark condition. **a** mass change curves of the evaporators. **b** evaporation rates of the evaporators.

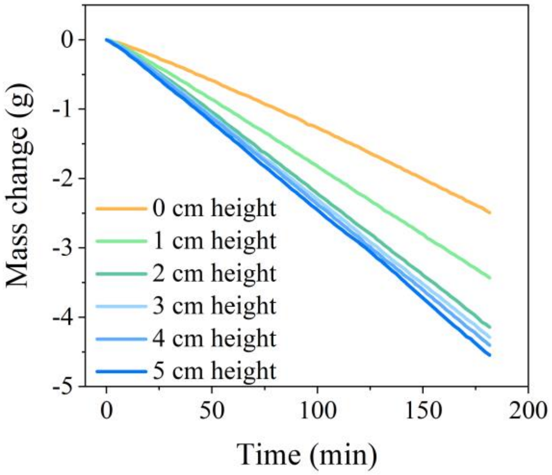

Figure S6. Mass change curves of evaporators with different heights under 1 sun illumination. In this experiment, deionized water is used for evaporation.

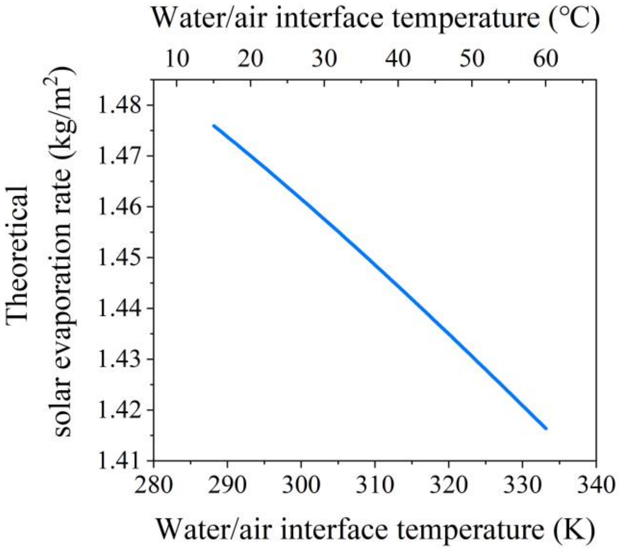

Figure S7. Theoretical solar evaporation rate by assuming 100% solar-to-vapor energy conversion efficiency.

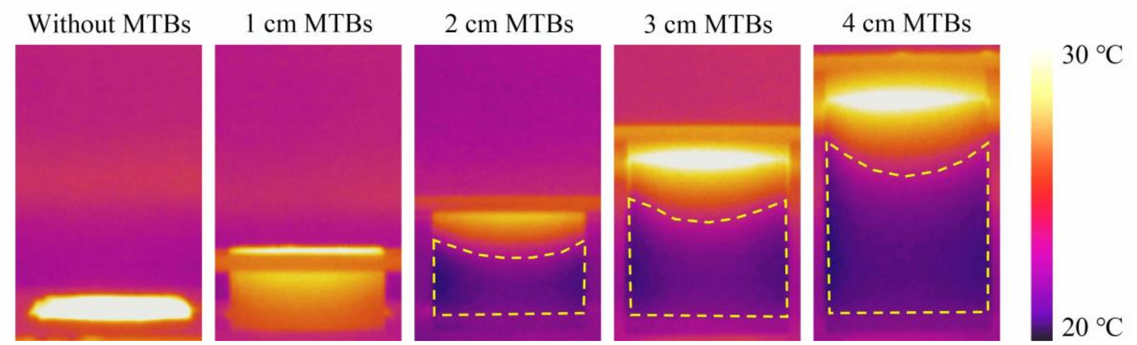

Figure S8. Side-view IR images of evaporators with different bridge heights. The evaporators are operated under 1 sun illumination dealing with deionized water. The temperature of circled areas is lower than room temperature, indicating the participation of natural evaporation in the overall vapor generation when the bridge height exceeds 2 cm.

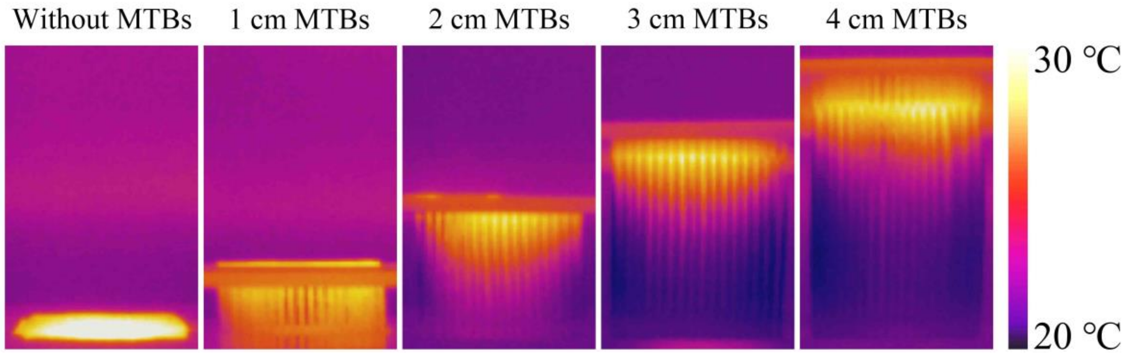

Figure S9. IR images of evaporators with different MTB heights during water evaporation under 1 sun illumination.

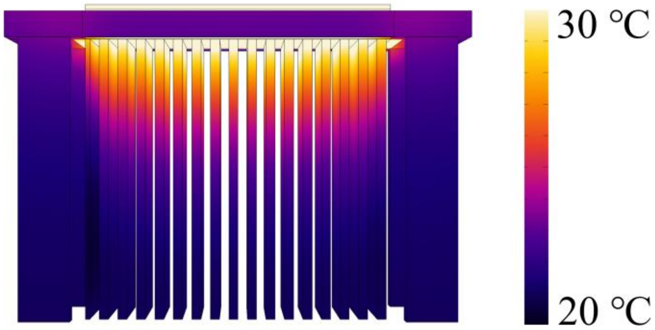

Figure S10. COMSOL simulation of the evaporator temperature distribution during deionized water evaporation under 1 sun illumination.

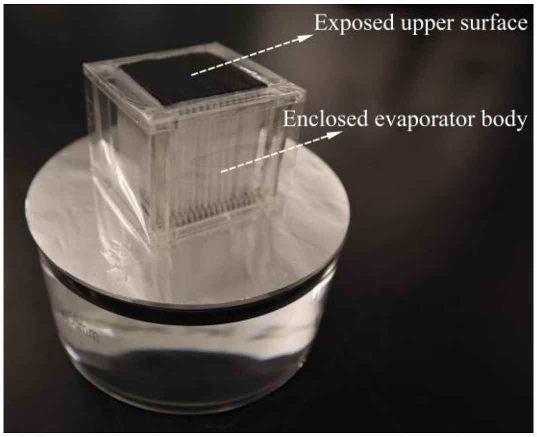

Figure S11. Photograph of the evaporator with an enclosed body. The evaporator body is wrapped by an airtight polypropylene film, leaving only the upper surface exposed to the surrounding environment to release vapor.

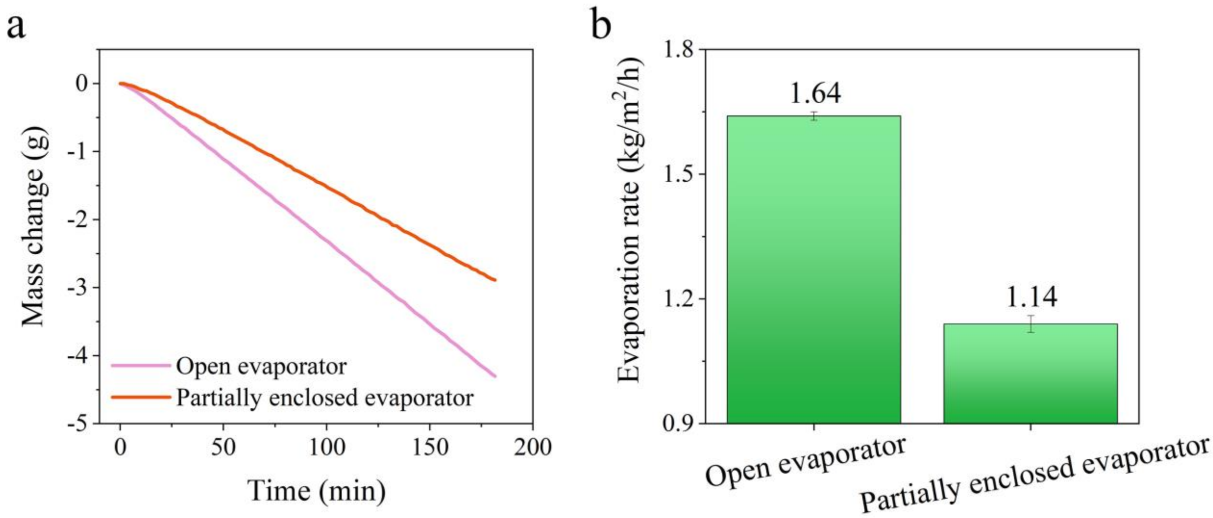

Figure S12. Evaporation performance of the open evaporator and the partially enclosed evaporator with only upper surface exposed. **a** Mass change curves of the evaporators during the tests. **b** The calculated evaporation rates.

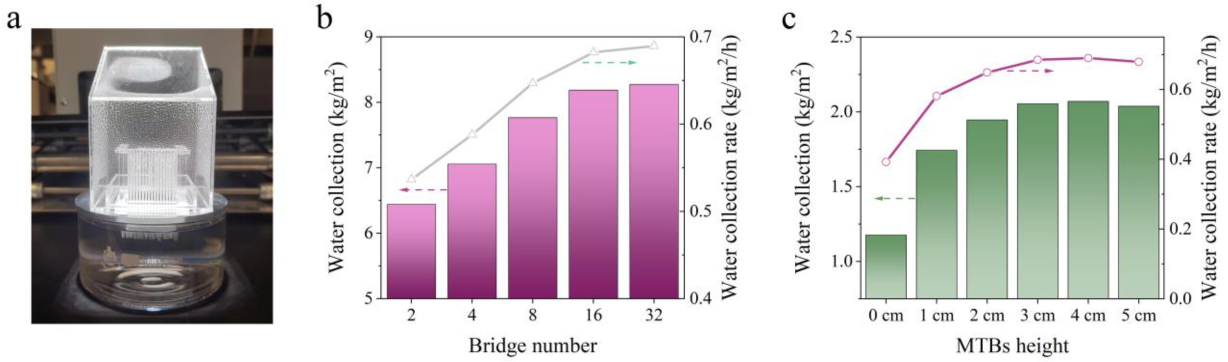

Figure S13. Water generation using an enclosed solar evaporation system. **a** photograph of the enclosed solar evaporation system for water collection. **b** the effect of the number of bridges on water generation, where the bridge height is fixed at 3 cm. **c** the effect of the height of bridges on water generation, where the bridge number is fixed at 32. The experiments were performed with 10 wt% NaCl solution under 1 sun illumination. In **b**, each test was performed for 12 hours. In **c**, each test was performed for 3 hours.

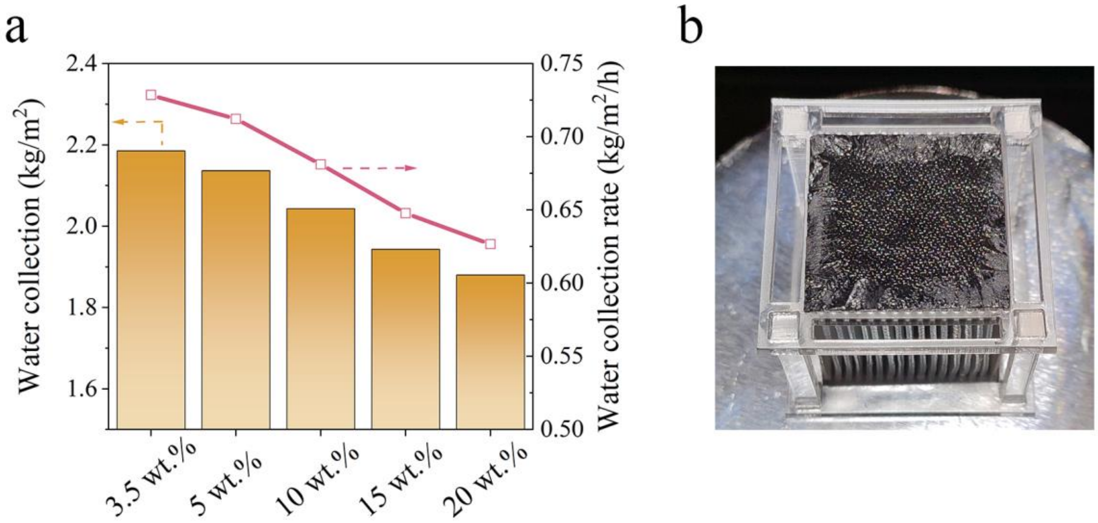

Figure S14. The influence of salinity on water generation. **a** water generation from brines of varying salinities (wt% of NaCl) using an enclosed solar evaporation system with an optimized MTB architecture. Each test was performed under 1 sun illumination for 3 hours. **b** photograph of the evaporator after 3 h of operation in 20 wt% brine, showing salt precipitation emerged at the periphery of the evaporator surface.

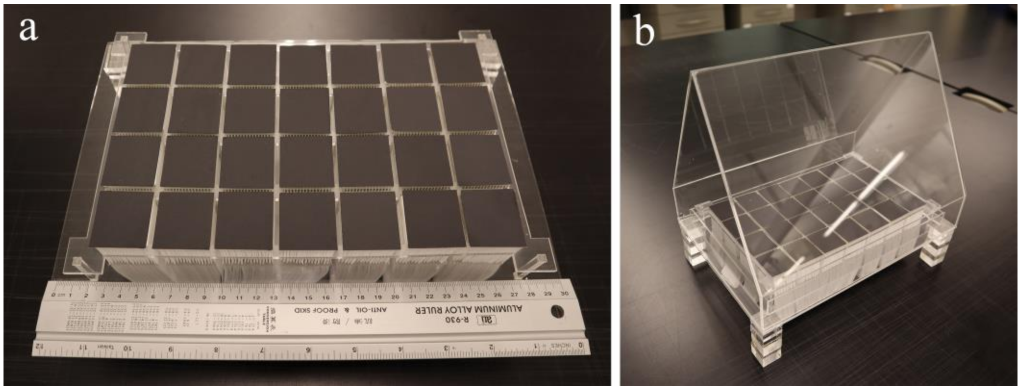

Figure S15. Photographs of the device for the outdoor experiments. **a** scaled-up evaporator. **b** evaporator equipped with a PMMA cover for practical water collection.

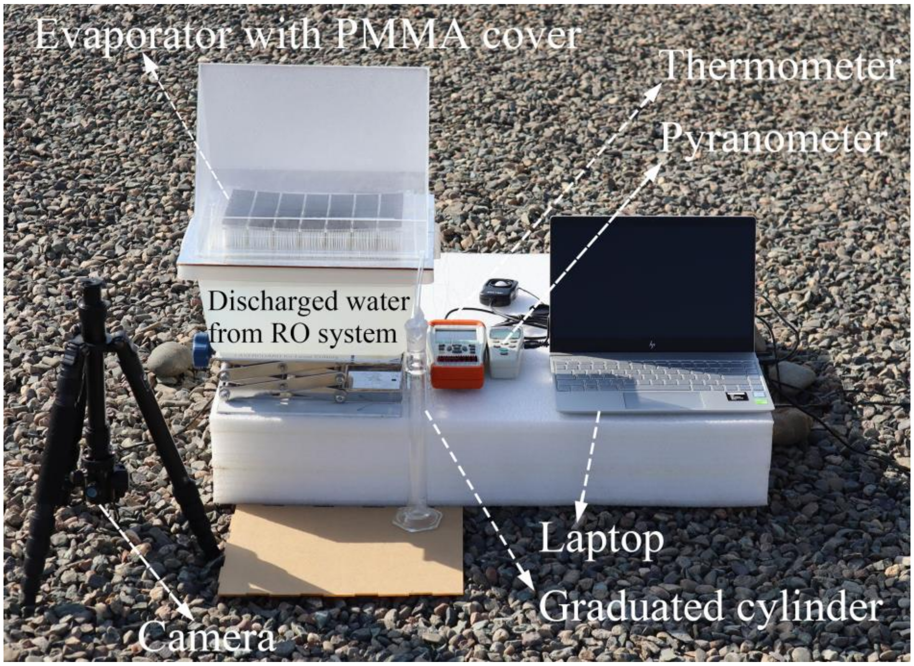

Figure S16. Photograph of the system setup for rooftop experiment.

Supplementary Notes S1

Theoretical limit of the solar evaporation rate

The theoretical solar evaporation limit (E_r) is calculated by assuming a 100% solar-to-vapor energy conversion efficiency as follows:

$$E_r = \frac{P_{solar}}{h_l} = \frac{P_{solar}}{Q_s + Q_l} = \frac{P_{solar}}{c(T_i - T_w) + 1.91845 \times 10^3 (T_i(T_i - 33.910))^2}$$

where P_{solar} (W/m^2/h) is the solar energy input; h_l (kJ/kg) is the total enthalpy required for the water evaporation, which is the summary of the sensible heat (Q_s) required for the temperature increase and the latent heat (Q_l) required for the phase change; Q_s (kJ/kg) is dependent on the water–air interface temperature (T_i, K) and the bulk water temperature (T_w, K); and Q_l (kJ/kg) is related to T_i. In our case, the bulk water temperature is 294.15 K and P_{solar} is 1000 W/m^2. Therefore, the theoretical evaporation rate is related to the water–air interface temperature. The calculated theoretical solar evaporation rate is shown in **Fig. S7**.

Supplementary Notes S2

Cost analysis

The materials used in this system include glass fiber membrane, carbon nanotube and acrylic plate. The cost of each materials is summarized below using same products found on Alibaba.com.

- ✓ Glass fiber membrane: 0.41 USD per square meter.
- ✓ Carbon nanotube: 2.9 USD per gram.
- ✓ Acrylic plate: 4.7 USD per square meter.

The fabrication of 1 m^2 solar evaporator requires ~28 m^2 glass fiber membrane, ~5 g carbon nanotubes, and ~4 m^2 acrylic plate, corresponding to a total material cost is ~44.8 USD.

The production cost of this 3D evaporator can be significantly reduced in large-scale manufacturing process by using low-cost alternative materials, such as carbon black and hydrophilic cotton fabric, to replace carbon nanotubes and glass fiber membrane, respectively.
