# Supplementary material for: Three-dimensional open architecture enabling salt-rejection solar evaporators with boosted water production efficiency
Source: Nat Commun. 2022 Nov 4;13:6653. doi: 10.1038/s41467-022-34528-7 (PMC9636182; doi:10.1038/s41467-022-34528-7)
Supplement: Supplementary file 3 — Description of Additional Supplementary Files [file 41467_2022_34528_MOESM3_ESM.pdf]

Description of Additional Supplementary Files

File Name: Supplementary Movie 1
Description: Time-lapse video showing the water collection process using a 3D solar evaporator with an evaporation area of 390 cm^2 in a field test. The water discharged from an RO system was used as the source water (salinity: ~8.7%), and the test lasted for nine hours from 8:00 am.
